# Advancing bone tumor detection in older adults: the impact of AI-enhanced medical imaging

**DOI:** 10.3389/fmed.2025.1697975

**Published:** 2025-11-19

**Authors:** Peng Liang, Yan Li, Peipei Feng, Sirong Wei

**Affiliations:** 1 Department of Imaging, Yantaishan Hospital, Yantai, Shangdong, China; 2 Department of Radiology, Yantai Qishan Hospital, Yantai, Shangdong, China; 3 Department of Vascular Intervention, Yantai Qishan Hospital, Yantai, Shangdong, China

**Keywords:** osteosarcoma, elderly, artificial intelligence, medical imaging, tumor detection, geriatric oncology

## Abstract

Osteosarcoma presents unique challenges in diagnosis and management, particularly among older adults, who often experience distinct clinical presentations and treatment complications. As the global demographic of individuals aged 65 and older continues to expand, the need for effective strategies to address osteosarcoma in this group becomes increasingly urgent. Medical imaging plays a major role in the detection and monitoring of bone tumors, yet traditional imaging approaches face significant challenges when applied to older adults, including age-related physiological changes and comorbidities. Recent advancements in Artificial Intelligence provide transformative potential to enhance medical imaging for osteosarcoma detection in older adults. AI-driven technologies can improve image acquisition, reduce artifacts, and automate tumor detection and segmentation, thereby increasing diagnostic accuracy and optimizing treatment strategies. This mini-review explores the critical role of AI-enhanced medical imaging in overcoming the unique challenges of diagnosing osteosarcoma in older adults, emphasizing the need for tailored algorithms and protocols that consider the specific anatomical and physiological characteristics of this vulnerable population.

## 1 Introduction

Osteosarcoma, a malignant bone tumor characterized by the production of osteoid matrix, is traditionally associated with younger populations, particularly adolescents and young adults (1). However, its occurrence in older adults presents unique challenges that demand specialized attention. The older adult population, defined as individuals aged 65 years and older, is rapidly growing worldwide, with projections indicating that it will constitute 16% of the global population by 2050 (2). This demographic shift has significant implications for healthcare systems, including the management of oncologic disorders such as osteosarcoma. In older adults, the presentation, progression, and treatment outcomes of osteosarcoma differ markedly from those observed in younger individuals, primarily due to age-related physiological changes, multimorbidity, and reduced tolerance to aggressive therapies (3).

The diagnosis and management of osteosarcoma in older adults are complicated by several factors. Age-related changes in bone structure, such as decreased bone density and altered marrow composition, can obscure imaging findings and mimic pathological processes (4). Furthermore, older adults often present with multiple comorbidities, including cardiovascular disease, diabetes, and neurodegenerative disorders, which complicate treatment planning and increase the risk of adverse outcomes (5). Polypharmacy and frailty further exacerbate these challenges, limiting the applicability of standard treatment protocols such as high-dose chemotherapy and surgical resection (6). Consequently, older adults with osteosarcoma frequently experience poorer prognoses and reduced survival rates compared to their younger counterparts (7).

Medical imaging plays an essential role in the detection, characterization, and monitoring of bone tumors, including osteosarcoma. Advanced modalities such as magnetic resonance imaging, computed tomography, and positron emission tomography are essential for delineating tumor extent, assessing treatment response, and identifying metastases (8). However, imaging older adults introduces additional complexities, including motion artifacts due to physical limitations, interference from implantable devices, and increased susceptibility to contrast-induced nephropathy (9). These challenges necessitate the development of tailored imaging protocols that account for the unique needs of the geriatric population.

Artificial intelligence (AI) has emerged as a transformative tool in medical imaging, offering unprecedented capabilities in image acquisition, interpretation, and analysis (10). Machine learning and deep learning algorithms have demonstrated remarkable proficiency in automating tumor detection, segmenting complex anatomical structures, and predicting treatment outcomes (11). Despite these advancements, the application of AI in bone tumor detection has predominantly focused on younger populations, with limited consideration of older adults. This gap in research is concerning, given the distinct physiological and pathological features of aging that can influence imaging findings and diagnostic accuracy.

The purpose of this mini-review is to explore how AI-enhanced medical imaging can address the unique challenges of bone tumor detection in older adults, with a specific focus on osteosarcoma. It also highlights the urgent need to develop AI models tailored to older adults, ensuring that advancements in medical imaging are equitably applied to improve diagnostic accuracy and optimize treatment strategies for this vulnerable group.

## 2 Osteosarcoma in older adults

### 2.1 Epidemiology and clinical presentation

Although osteosarcoma is relatively rare in older adults, its occurrence is often linked to secondary causes such as pre-existing bone conditions or prior radiation therapy. Older adults frequently present with atypical tumor locations, such as flat bones (e.g., pelvis or scapula), rather than the long bones typically affected in younger individuals (12). Furthermore, the clinical presentation in older adults is often subtle, with symptoms such as localized pain, swelling, or reduced mobility being attributed to age-related musculoskeletal degeneration, leading to delays in diagnosis (13).

### 2.2 Pathological features

The biological behavior of osteosarcoma in older adults is distinct from that in younger populations. Tumors in older adults tend to exhibit slower growth rates and a higher degree of differentiation, yet they are often more aggressive due to delayed detection and advanced disease at presentation (14). Histopathological analysis frequently reveals a predominance of osteoblastic subtypes, but mixed or fibroblastic variants may also be observed (15). Age-related changes in bone marrow composition, including increased yellow marrow and reduced vascularity, can influence tumor growth dynamics and complicate imaging interpretation (16).

### 2.3 Challenges in treatment

The treatment of osteosarcoma in older adults is inherently complex due to physiological changes associated with aging, multimorbidity, and reduced tolerance to aggressive therapies (17). Standard treatment protocols, including neoadjuvant chemotherapy, surgical resection, and adjuvant chemotherapy, are often less effective or poorly tolerated in older adults. Age-related reductions in renal and hepatic function compromise the metabolism and clearance of chemotherapeutic agents, increasing the risk of toxicity. Furthermore, older adults with osteosarcoma frequently exhibit frailty, defined as a multidimensional syndrome of decreased physiological reserve, which limits their ability to withstand invasive procedures and systemic therapies (18).

Radiotherapy, often considered an adjunctive treatment for osteosarcoma, poses additional challenges in older adults due to increased susceptibility to radiation-induced complications, such as fractures and soft tissue necrosis (19). All together, these factors contribute to poorer treatment outcomes and reduced survival rates in older adults with osteosarcoma compared to their younger counterparts.

## 3 Medical imaging in osteosarcoma with special considerations in older adults

Medical imaging is an indispensable tool in musculoskeletal oncology, serving as the basis for the diagnosis, staging, treatment planning, and monitoring of bone tumors such as osteosarcoma. Advanced imaging modalities, including magnetic resonance imaging (MRI), computed tomography (CT), positron emission tomography (PET), and ultrasound, are pivotal in providing detailed anatomical and functional information that guides clinical decision-making. However, the application of these modalities in older adults presents unique challenges that stem from the physiological changes associated with aging, the presence of comorbid conditions, and physical limitations. These factors demand a tailored approach to imaging that addresses the specific needs of this vulnerable population.

### 3.1 Challenges in imaging older adults

Imaging older adults with musculoskeletal tumors presents unique challenges due to age-related anatomical changes, physical and cognitive limitations, and the presence of comorbidities. Aging alters bone structure, including the natural progression of red-to-yellow marrow conversion, which can obscure imaging findings and mimic pathological processes. For example, marrow reconversion due to stress or pathology may resemble metastatic disease on MRI, complicating differentiation between normal aging and malignancy (20). Furthermore, osteoporosis, prevalent in older adults, increases the likelihood of fractures, which can mimic malignant compression fractures (18). Calcifications in vasculature and soft tissues, common in older individuals, further obscure tumor margins or mimic tumor-related findings, particularly on CT scans (21).

Physical and cognitive limitations also interfere with imaging quality in older adults. Tremors, arthritis, and cognitive impairments such as dementia often result in motion artifacts that degrade image clarity, necessitating repeat scans and increasing patient discomfort (22). Implantable devices like pacemakers and joint prostheses create artifacts on MRI and CT scans, obscuring critical anatomical details. In addition, contrast media present risks for older adults with reduced renal function, as they are more susceptible to contrast-induced nephropathy (23). While non-contrast imaging modalities are safer, they may compromise diagnostic sensitivity, creating a trade-off between accuracy and patient safety.

Also, multimorbidity complicates imaging interpretation in older adults. Conditions such as diabetes, cardiovascular disease, and chronic kidney disease can produce secondary changes that mimic or obscure tumor-related pathology (24). For example, diabetic bone changes or vascular calcifications may resemble tumor involvement, leading to diagnostic uncertainty. These complexities highlight the need for geriatric-specific imaging considerations and multidisciplinary collaboration among radiologists, oncologists, and geriatricians to ensure accurate diagnosis and effective treatment planning for older adults with musculoskeletal tumors.

### 3.2 Imaging modalities and their challenges

Among the various imaging modalities available, each has specific strengths and limitations in the evaluation of musculoskeletal tumors in older adults. MRI is often the modality of choice for assessing bone marrow and soft tissue involvement in osteosarcoma, providing high-resolution images that are critical for evaluating tumor extent, vascular invasion, and treatment response. However, MRI is not without challenges in elderly populations, as implant interference, tissue heating risks, and acoustic injury in patients with hearing loss can limit its applicability (25). CT is particularly valuable for detecting cortical bone destruction, evaluating pathological fractures, and identifying pulmonary metastases, which are common in osteosarcoma (26). Despite its utility, metal artifacts from implants remain a significant limitation in older adults undergoing CT imaging. PET, often combined with CT (PET-CT), is highly sensitive for detecting metastatic disease and monitoring treatment response, making it an essential tool in the management of advanced osteosarcoma (27). However, the prolonged immobilization required during PET scans can be difficult for older adults to tolerate, reducing the feasibility of this modality in some cases. Ultrasound, while less commonly used for musculoskeletal tumors, remains a valuable non-invasive and cost-effective tool for evaluating superficial lesions and guiding biopsies, particularly in older adults who may not tolerate more invasive procedures.

### 3.3 Optimizing imaging protocols for older adults

To address these challenges, specific adjustments and strategies can be used to optimize imaging outcomes for older adults. Tailored imaging protocols that minimize patient discomfort and reduce scan times are essential, particularly for modalities like MRI and PET that require prolonged immobilization (28). Pre-imaging preparation strategies, such as the use of shallow breathing techniques, light indicators for hearing-impaired patients, or sedation for those with severe cognitive impairments, can improve the success rates of imaging studies while enhancing patient comfort. Multimodality approaches that combine the strengths of different imaging techniques, such as MRI and CT, can provide complementary information and reduce diagnostic ambiguity, ensuring a more comprehensive evaluation of musculoskeletal tumors (29).

### 3.4 Recent advances in imaging technology

Recent advancements in imaging technology offer promising solutions to many of the challenges associated with imaging older adults. Among them, AI has revolutionized medical imaging by enhancing image acquisition, reducing artifacts, and automating bone tumor detection (30, 31). AI algorithms are increasingly being integrated into imaging workflows to improve diagnostic accuracy and streamline processes, offering significant potential for addressing the unique challenges of imaging older adults with musculoskeletal tumors. The transformative impact of AI in this context will be explored in more detail in the next section.

Table 1 provides an overview of current AI models relevant to osteosarcoma detection, highlighting their open-source availability and key functionalities. The models range from deep learning approaches designed for automated tumor detection in MRI images to convolutional neural networks focused on predicting bone tumors from X-ray images. In addition, it includes a machine learning model for classifying osteosarcoma histological images and a hybrid deep learning approach that integrates multiple imaging modalities to enhance detection accuracy. Notably, while there is ongoing development in AI-based reconstruction techniques aimed at reducing motion artifacts in MRI, such advancements have yet to be specifically tailored for osteosarcoma. Nevertheless, we have included it in the table to showcase a potential future development for osteosarcoma. This summary highlights the diverse applications of AI in improving diagnostic capabilities for osteosarcoma and points to areas where further research and development are needed.

**Table 1:** Examples of existing AI models relevant to osteosarcoma detection.

**Model**	**Open source status**	**Description**	**References**
Deep learning	Yes	Automated tumor detection using MRI images.	(46, 47)
Convolutional neural network (CNN)	Yes	Bone tumor prediction using CT and MRI images	(41, 48)
Machine learning	Yes	Classification of osteosarcoma histological images	(49, 50)
Hybrid deep learning	No	Integrates multiple imaging modalities for enhanced osteosarcoma detection	(51, 52)
AI-based reconstruction (not yet developed for osteosarcoma)	Yes	Reduces motion artifacts in MRI images	(53, 54)

This table lists some AI models, their open-source status, links to their respective repositories, and a brief description of their functionalities in the context of osteosarcoma detection and imaging.

## 4 Artificial intelligence in medical imaging: transforming osteosarcoma diagnosis in older adults

While much of the research and application of AI in musculoskeletal oncology has focused on younger populations (32–34), its integration into imaging workflows for older adults remains underexplored. This section discusses the potential of AI to address the unique challenges posed by osteosarcoma in older adults, highlighting its role in improving diagnostic precision, optimizing treatment strategies, and advancing personalized care.

### 4.1 AI-driven enhancements in image acquisition and processing

AI provides significant advancements in image acquisition and processing, addressing many of the limitations inherent in imaging older adults. For example, motion artifacts, which are common in older individuals due to physical or cognitive impairments, can be mitigated through AI-based reconstruction algorithms (35). These algorithms analyze incomplete or distorted data and generate high-quality images, reducing the need for repeat scans and minimizing patient discomfort. Similarly, AI-based denoising techniques improve image clarity by suppressing artifacts caused by implantable devices, such as pacemakers or joint prostheses (36), which are prevalent in older adults. These enhancements not only improve diagnostic accuracy but also ensure that imaging studies are more efficient and patient-friendly.

Photon-counting CT, an emerging technology integrated with AI, exemplifies the transformative potential of AI in image acquisition. This modality provides superior resolution and contrast efficiency, allowing radiologists to differentiate between benign age-related changes, such as osteoporosis, and malignant bone lesions like osteosarcoma (37). Integrating AI with advanced imaging technologies allows clinicians to overcome the diagnostic ambiguities associated with aging physiology and comorbidities, leading to more precise assessments of tumor extent and progression.

### 4.2 Tumor detection and segmentation

One of the most impactful applications of AI in medical imaging is its ability to automate tumor detection and segmentation (38). Deep learning algorithms, particularly convolutional neural networks, excel at identifying subtle abnormalities in imaging studies that may be overlooked by human observers (39). For older adults with osteosarcoma, where age-related changes in bone structure and marrow composition complicate tumor visualization, AI offers unparalleled sensitivity and specificity. By training algorithms on large datasets that include imaging studies from elderly populations, AI systems can learn to recognize the distinct features of osteosarcoma in this demographic, accounting for variations in anatomy and pathology.

Segmentation, the process of delineating tumor boundaries, is another critical task that benefits from AI integration (40). Accurate segmentation is essential for assessing tumor size, evaluating invasion into surrounding tissues, and planning surgical resection or radiotherapy. AI algorithms automate this process with remarkable precision, reducing interobserver variability and ensuring consistent results (41). For older adults, whose imaging studies often include confounding factors such as vascular calcifications or degenerative changes, AI-driven segmentation provides a reliable means of distinguishing tumor margins from background noise, facilitating more effective treatment planning.

### 4.3 AI in monitoring and longitudinal care

AI may also transform the monitoring and follow-up of older adults with osteosarcoma. Traditional imaging methods for assessing treatment response and detecting recurrence often rely on subjective interpretation, which can vary among radiologists and may be influenced by age-related changes in imaging findings. AI algorithms overcome these limitations by quantifying changes in tumor size, density, and metabolic activity over time, providing objective metrics that enhance the accuracy of longitudinal assessments (42).

For older adults, who are at higher risk of complications such as pathological fractures or radiation-induced changes, AI can identify early signs of adverse events, enabling timely interventions. Furthermore, AI-powered imaging platforms can integrate data from multiple modalities, such as MRI, CT, and PET, creating comprehensive profiles of tumor progression and treatment response (43). This holistic approach ensures that clinicians have a complete understanding of the patient's condition, facilitating proactive management and improving long-term outcomes.

### 4.4 Challenges and ethical considerations

Despite its transformative potential, the integration of AI into medical imaging for older adults with osteosarcoma is not without challenges. One of the primary obstacles is the lack of large, high-quality datasets that represent the unique imaging characteristics of older adults. Most AI models are trained on datasets derived from younger individuals, limiting their applicability to older adults with distinct anatomical and pathological features (44). Addressing this gap requires the development of geriatric-specific datasets and the inclusion of older adults in AI research initiatives.

Ethical considerations also play a critical role in the adoption of AI in medical imaging (45). For older adults, who may have limited understanding of AI technologies, ensuring transparency and informed consent is essential. Clinicians must communicate the benefits and limitations of AI applications in a way that is accessible and reassuring, fostering trust and confidence in these advancements. In addition, the use of AI raises concerns about data privacy and security, particularly for vulnerable populations such as older adults. Robust safeguards must be implemented to protect patient information and ensure compliance with ethical standards.

## 5 Recommendations and future research directions

As previously discussed, several gaps remain in the application of AI and related imaging technologies to the older adults. This section outlines key recommendations and future research directions aimed at addressing these challenges and advancing care for older adults with osteosarcoma.

To optimize the use of AI and improve outcomes for older adults, some actionable recommendations can be implemented:

### 5.1 Develop AI algorithms tailored for older adults

Current AI models primarily rely on datasets from younger populations, which limits their effectiveness for older adults. Therefore, it is of primary importance to create AI algorithms specifically designed for older adults with musculoskeletal tumors. These algorithms should incorporate age-specific imaging data that reflects the anatomical and physiological changes associated with aging, such as osteoporosis, vascular calcifications, and altered marrow composition. This tailored approach will enhance the accuracy of tumor detection and segmentation in older populations.

Furthermore, there is a pressing need for research that evaluates AI performance in older adults with musculoskeletal tumors. Studies should focus on validating these algorithms with datasets that accurately represent older adults, ensuring that they account for age-related anatomical and pathological variations. Comparative research should also investigate the impact of AI-enhanced imaging on improving diagnostic accuracy and treatment outcomes for this demographic.

### 5.2 Enhance multidisciplinary collaboration

Effective management of older adults with osteosarcoma requires collaboration between radiologists, oncologists, geriatricians, and other specialists. AI-enhanced imaging technologies can serve as a unifying platform for multidisciplinary teams, facilitating shared decision-making and improving diagnostic accuracy. Training programs and workshops focused on AI applications in geriatric oncology should be implemented to foster collaboration and ensure that healthcare professionals are equipped to leverage these advancements.

**Figure 1:**
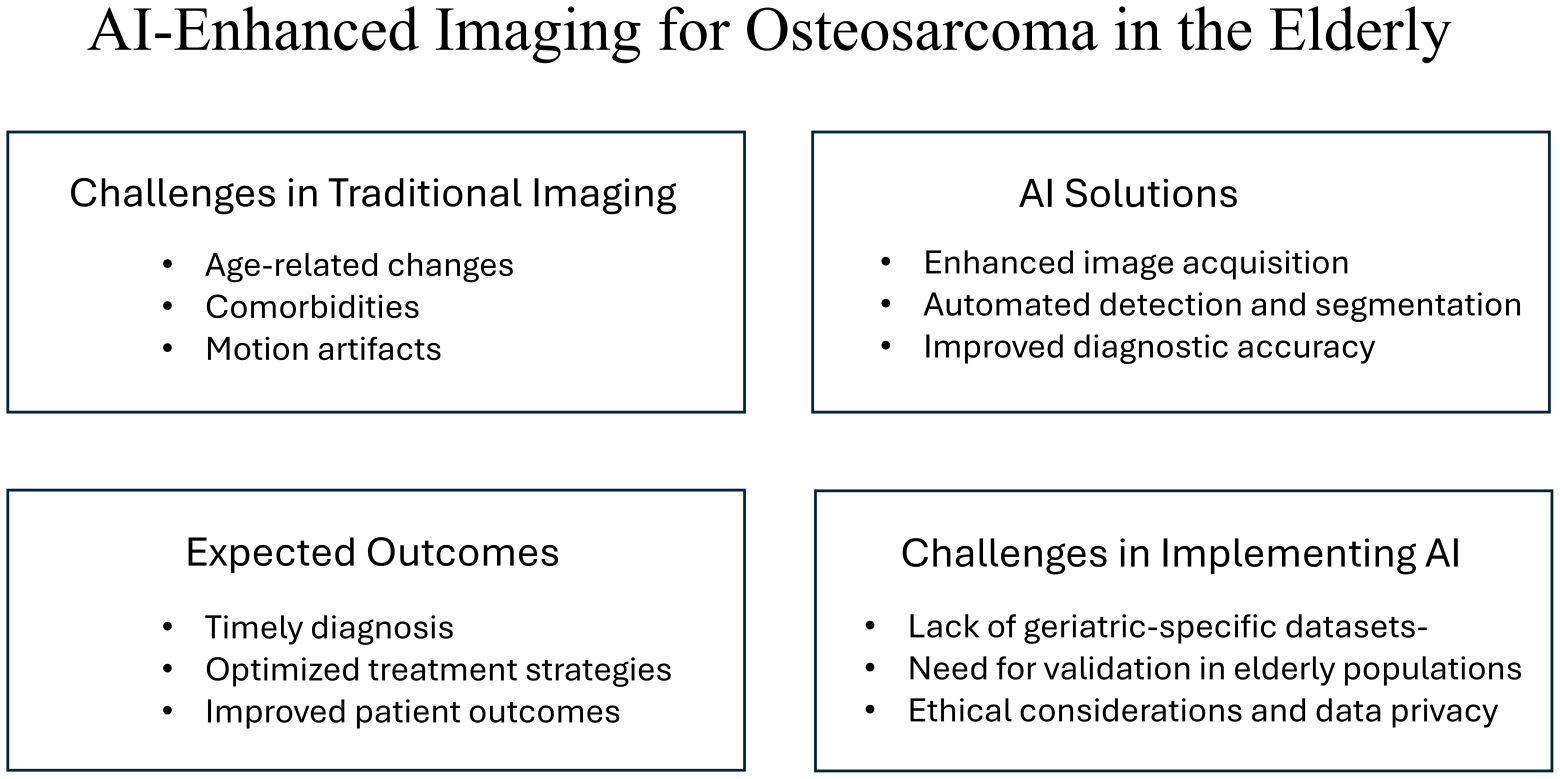
Overview of AI-enhanced imaging for osteosarcoma detection in older adults. The figure highlights the challenges associated with traditional imaging, the potential solutions provided by AI technologies, the expected outcomes from their implementation, and the specific challenges faced in integrating AI into clinical practice for this demographic.

Figure 1 summarizes the key messages of this mini-review and illustrates the multifaceted approach of AI-enhanced imaging in addressing the unique challenges of osteosarcoma detection in older adults.

## 6 Conclusion

In conclusion, the integration of artificial intelligence into medical imaging holds significant promise for improving the detection and management of osteosarcoma in older adults. As this demographic faces unique challenges, AI-enhanced imaging technologies can provide crucial support in overcoming these barriers. Future research should focus on developing AI algorithms specifically designed for the elderly population, ensuring that advancements in medical imaging are effectively used to address their unique challenges. By fostering interdisciplinary collaboration among radiologists, oncologists, and geriatric specialists, we can harness the full potential of AI to improve outcomes for older adults with osteosarcoma, ultimately leading to better care and quality of life for this vulnerable group.
